# Hydroxytyrosol Ameliorates Intervertebral Disc Degeneration and Neuropathic Pain by Reducing Oxidative Stress and Inflammation

**DOI:** 10.1155/2022/2240894

**Published:** 2022-11-02

**Authors:** Haichao Yu, Zhen Zhang, Fahong Wei, Guowei Hou, Yonggang You, Xiangyu Wang, Shiqi Cao, Xiaoqing Yang, Weibo Liu, Shaofu Zhang, Fanqi Hu, Xuesong Zhang

**Affiliations:** ^1^School of Medicine, Nankai University, Tianjin, China; ^2^Department of Orthopedics, The Fourth Medical Center, Chinese People's Liberation Army General Hospital, Beijing, China; ^3^Medical School of Chinese People's Liberation Army General Hospital, Beijing, China; ^4^Department of Clinical Laboratory, Linyi Central Hospital, Linyi, Shandong, China; ^5^Department of Orthopedics, PKU Care Luzhong Hospital, Zibo, Shandong, China; ^6^Department of Orthopedics, 926th Hospital of Joint Logistic Support Force, The Affiliated Hospital of Kunming University of Science and Technology, Yunnan, China; ^7^Department of Pain Medicine, The First Medical Center, Chinese People's Liberation Army General Hospital, Beijing, China

## Abstract

Low back pain (LBP) seriously affects human quality of life. Intervertebral disc degeneration (IVDD) is the main pathological factor that leads to LBP, but the pathological mechanism underlying IVDD has not been fully elucidated. Neuropathic pain caused by IVDD is an important pathological factor affecting people's daily lives. Therefore, it is very important to identify therapeutic drugs to ameliorate IVDD and secondary neuropathic pain. Hydroxytyrosol (HT) is a natural compound derived from olive leaves and oil and has anti-inflammatory, antioxidant, and antitumor activities and other properties. In this study, TNF-*α*-stimulated human nucleus pulposus cells (HNPCs) were used to simulate the local inflammatory microenvironment observed in IVDD in vitro to explore the role of HT in alleviating various pathological processes associated with IVDD. A rat needle puncture model was used to further explore the role of HT in alleviating IVDD. Lipopolysaccharide (LPS) was used to stimulate microglia in vitro to comprehensively explore the role of HT in alleviating neuropathic pain, and a rat model involving chronic compression of the dorsal root ganglion (CCD) was established to simulate the neuropathic pain caused by IVDD. This study suggests that HT reduces the expression of cyclooxygenase-2 (COX-2), inducible nitric oxide synthase (iNOS), the NOD-like receptor thermal protein domain associated protein 3 (NLRP3) inflammasome, a disintegrin and metalloproteinase with thrombospondin motifs-4 (ADAMTS-4) and matrix metalloproteinase-13 (MMP-13); inhibits the production of mitochondrial reactive oxygen species (ROS); and maintains mitochondrial homeostasis. Thus, HT appears to reduce the rate of apoptosis and mitigate the loss of major intervertebral disc components by inhibiting the nuclear factor kappa-B (NF-*κ*B) signaling pathway. Moreover, HT inhibited the secretion of COX-2, tumor necrosis factor-*α* (TNF-*α*), interleukin (IL)-6, IL-1*β*, and iNOS and activation of the NLRP3 inflammasome in microglia by inhibiting the phosphatidylinositol 3-kinase (PI3K)/protein kinase B (AKT) and extracellular regulated protein kinase (ERK) signaling pathways. In conclusion, HT plays a protective role against IVDD and secondary neuropathic pain by inhibiting the NF-*κ*B, PI3K/AKT, and ERK signaling pathways.

## 1. Introduction

Low back pain (LBP) is a common symptom in orthopedic patients. Research suggests that up to 84% of the global population will experience LBP in their lifetime [1, 2]. Intervertebral disc degeneration (IVDD) is the main pathological factor that causes LBP [3, 4]. The treatment of IVDD remains a controversial topic; treatment options include rest, pain medication, physical therapy, and surgery [5]. These methods are designed to relieve the patient's pain but not to repair the damaged intervertebral disc (IVD). IVDD is an extremely complex pathological process. Abnormal mechanical stress, sudden trauma, bacterial infection, smoking, aging, and other pathogenic factors lead to the increased expression of inflammatory cytokines (such as interleukin (IL)-1*β*, IL-6, IL-17, and tumor necrosis factor-*α* (TNF-*α*)) in nucleus pulposus (NP) cells. The release of inflammatory cytokines leads to an increase in the expression of major catabolic enzymes (a disintegrin and metalloproteinase with thrombospondin motifs-4, 5 (ADAMTS-4, 5) and matrix metalloproteinase-13 (MMP-13)) in NP cells, resulting in the degradation of the extracellular matrix (ECM) and an imbalance between catabolic and anabolic metabolism in NP cells and the occurrence of IVDD [6].

Treatment of NP cells with TNF-*α*, a traditional inflammatory cytokine, has been widely used to establish IVDD models in vitro [7]. TNF-*α* triggers many pathological processes involved in IVDD through the activation of the NF-*κ*B signaling pathway, including the secretion of a variety of proinflammatory cytokines (IL-1*β*, IL-6, and IL-8), the production of MMPs and ADAMTSs that promote ECM degradation, increased decomposition of collagen-2 (col-2), and aggrecan, and damage to the structure of IVDs. TNF-*α* exacerbates IVDD by activating the NOD-like receptor thermal protein domain associated protein 3 (NLRP3) inflammasome, thus mediating mitochondrial dysfunction and reactive oxygen species (ROS) production in NP cells [7].

Mitochondria are the main organelles producing ROS [8]. Mitochondria are the target of ROS, and the accumulation of ROS alters cellular metabolism, causing oxidative damage to IVD cells and leading to the activation of the NLRP3 inflammasome and the release of inflammatory cytokines [9]. The overproduction of ROS substantially inhibits matrix synthesis and upregulates the expression of proteases that cause matrix degradation in IVD cells [10]. ROS can cause oxidative damage to mtDNA and respiratory enzymes, resulting in the imbalance and dysfunction of mitochondrial energy metabolism and thus leading to apoptosis and the exacerbation of IVDD. Therefore, reducing the inflammatory response in the local microenvironment of IVDs, improving mitochondrial function and inhibiting the activation of the NLRP3 inflammasome are important strategies to delay IVDD progression.

The occurrence of neuropathic pain seriously affects patient quality of life and makes treatment difficult [11]. Neuropathic pain is caused by damage to the peripheral or central nervous system and is characterized by spontaneous pain and hyperalgesia in response to innocuous and noxious stimulation [12–14]. The exacerbation of IVDD leads to annulus fibrosus (AF) tears and NP herniation. When disc degeneration results in disc herniation, compression of adjacent nervous system structures, such as nerve roots or dorsal root ganglia, occurs; this compression causes peripheral nerve damage and aggravates neuropathic pain, since the damaged sensory neurons produce inflammatory cytokines and chemokines that activate microglia and astrocytes [15, 16].

Inflammatory cytokines are important initiators and aggravators of neuropathic pain. Accumulating evidence suggests that proinflammatory cytokines (IL-1*β*, IL-6, cyclooxygenase-2 (COX-2), inducible nitric oxide synthase (iNOS), and TNF-*α*) are strongly involved in the pathogenesis of neuropathic pain [17–19]. In addition, the NLRP3 inflammasome has been widely reported to promote neuroinflammatory responses, leading to the worsening of neuropathic pain [20–22]. Proinflammatory cytokines are upregulated in injured peripheral nerves and causes chronic neuroinflammation while activating microglia and astrocytes in the spinal dorsal horn (SDH), which play key roles in neuropathic pain [23]. Microglia are necessary for the maintenance, support, protection, and monitoring of the central nervous system [24]. Substantial microglial hyperplasia in the SDH leads to local inflammatory responses that aggravate neuropathic pain [18, 25]. Microglia interact with neurons related to pain transmission and increase the excitability of these neurons, leading to the occurrence and progression of neuropathic pain [26]. Therefore, inhibiting the inflammatory response is an important way to alleviate neuropathic pain. Chronic compression of the dorsal root ganglion (CCD) is one of the important methods used to study neuropathic pain, and it has become an important disease model to simulate the neuropathic pain caused by disc herniation and spinal canal stenosis due to its direct compression of the dorsal root ganglion [16]. Therefore, we established a rat model of CCD to simulate local nerve compression caused by disc herniation and to study the mechanism by which hydroxytyrosol (HT) alleviates neuropathic pain.

HT is a natural phenol and the most biologically active component in olive leaves and olive oil. HT has multiple biological effects, and its anti-inflammatory and antioxidant effects have been widely studied. HT exerts its strong anti-inflammatory effects by inhibiting the expression of lipopolysaccharide (LPS)-mediated inflammatory cytokines (TNF-*α* and IL-1*β*) [27] and by inhibiting the expression of MMP-9 and COX-2 in activated human monocytes [28]. Among the various phenols in olives, HT has the strongest antioxidant activity due to its abilities to supply electrons in the ortho position of its hydroxyl group and to form stable hydrogen bonds with the phenoxy radical [27, 29]. Various studies have demonstrated that HT has therapeutic effects in cancer, neurodegenerative diseases, rheumatoid arthritis, osteoarthritis, and osteoporosis [30–33]. Previous studies have shown that HT-20 can inhibit acute inflammation and hyperalgesia induced by carrageenan in rats and reduce the local expression levels of IL-1*β* and TNF-*α* in rat tissues [34]. In a double-blind clinical trial, HT led to significantly better Japanese Orthopaedic Association (JOA) scores and visual analog scale scores than placebo in patients, demonstrating that HT is effective in reducing pain in gonarthrosis [35]. Moreover, HT inhibits several important pathological processes related to the occurrence and development of osteoarthritis; for example, HT reduces the production of important inflammatory cytokines, improves the local metabolic microenvironment, and inhibits the oxidative stress response [32, 36]. However, the mechanisms by which HT alleviates IVDD and neuropathic pain have not been reported. In the present study, we investigated the mechanisms by which HT alleviates neuropathic pain and IVDD.

## 2. Materials and Methods

### 2.1. Ethics Statement and Human Nucleus Pulposus Cell (HNPC) Extraction

The IVD tissues of 7 patients with IVDD were collected at Chinese PLA General Hospital (Beijing, China). The extraction of primary HNPCs was carried out according to a previously published study [37]. Briefly, NP tissues were cut into 1 mm^3^ pieces and digested with trypsin and type II collagenase. After 4 h of digestion, we used a 70 *μ*m sterile cell filter to filter the digested HNPCs. The HNPCs were washed three times to remove the remaining type II collagenase and then seeded in petri dishes [37].

### 2.2. HNPC and Microglia Culture

HNPCs were divided into four groups and cultured under different conditions: PBS, TNF-*α* (50 ng/mL) (ABclonal, China), TNF-*α* + HT (20 *μ*M) (MedChemExpress, China), and TNF-*α* + HT (100 *μ*M). Rat microglia (Procell Life Science & Technology Co., Ltd.) were divided into four groups and cultured under different conditions according to a previous study [38]: PBS, LPS (1 *μ*g/mL) (PeproTech, USA), LPS + HT (20 *μ*M), and LPS + HT (100 *μ*M).

### 2.3. Cell Viability

Cell viability was determined using the CCK-8 assay (Dojindo, Japan) following the manufacturer's instructions. The HNPC suspension (10^4^ cells) was added to 96-well plates. The culture plates were placed in an incubator for preculture. Ten microliters of CCK-8 solution was added to each well, and the cells were incubated for 2 h. The absorbance at 450 nm was then measured with a microplate reader.

### 2.4. Rats

Two-month-old Sprague–Dawley rats were obtained from Beijing Vitalstar Biotechnology Co., Ltd. All rats were randomly assigned to 3 groups (*N* = 5 per group). After successfully anesthetizing the rats, we used X-ray to assess the Co6/7 intervertebral space of the rats. A 20 G puncture needle was used to penetrate the AF of the IVD to the center of the NP, rotated 360°, and then removed after one minute. Two microliters of HT (100 *μ*M) was injected slowly for approximately 8 seconds using a Hamilton microsyringe with a 33 G needle on day 2 after the IVDD model was established.

All rats were randomly assigned to 4 groups (*N* = 5 per group). Two-month-old SD rats were anesthetized, the right paravertebral muscle of the L4-5 segment of rats was separated to expose the right lamina and the outer edge of the lamina, and L-shaped titanium rods (approximately 4 mm at one end and 3 mm at the other end) with a diameter of 0.63 mm were inserted into the L4 and L5 foramen. The rods were inserted into the foramen at 30° to the dorsal midline and 10° to the vertebral horizontal line [16]. In the sham group, only the L4 and L5 laminae and the outer edge of the lamina were exposed. The rats in the CCD model group (*N* = 5) were treated with intrathecal injection on the second day after the operation, and 10 *μ*L HT (100 *μ*m) was injected slowly for approximately 40 seconds.

### 2.5. Assessment of Pain Behaviors

Three researchers assessed the rats' behavioral scores separately and in a double-blind manner. As previously reported [39], beginning on Day 0 postoperatively, the paw withdrawal mechanical threshold (PWMT) was assessed by the same researcher every two days using the BME-404 electronic mechanical pain detector (Chinese Academy of Medical Sciences, CAMS, Beijing, China), and thermal paw withdrawal latency (TPWL) was assessed by the same researcher every two days using the BME-410C thermal analgesia tester (CAMS).

### 2.6. Quantitative Real-Time PCR (qRT–PCR)

HNPCs were incubated for 24 h with 50 ng/mL TNF-*α* and 20 or 100 *μ*M HT or were left untreated. Microglia were incubated for 24 h with 1 *μ*g/mL LPS and 20 or 100 *μ*M HT or were left untreated. Total RNA was extracted from HNPCs and microglia in each group with an RNA extraction kit (Yishan Biotechnology, China) and reverse transcribed into cDNA with a 20 *μ*L reverse transcription kit (Yishan Biotechnology). 2 × RealStar Power SYBR Real-time Quantitative PCR Mix (High ROX) (Genstar, China) on a 7500 RT–PCR system (ABI, USA) was used to conduct qRT–PCR.  Table 1 lists all the nucleotide sequences of the primers used in this experiment.

### 2.7. Western Blotting (WB) Analysis

HNPCs were incubated for 1 h or 48 h with 50 ng/mL TNF-*α* and 20 or 100 *μ*M HT or were left untreated. Microglia were incubated for 1 h or 48 h with 1 *μ*g/mL LPS and 20 or 100 *μ*M HT or were left untreated. The SDH was collected from the rats and lysed for 1 h with 100-150 *μ*L protein extraction buffer (1% PMSF+RIPA); the total protein contents in microglia and HNPCs were then extracted. The proteins were separated and transferred to polyvinylidene fluoride (PVDF) membranes. The bands were incubated with primary antibodies (all at 1 : 1000 dilution), including antibodies targeting NLRP3, COX-2, MMP-13, iNOS, ADAMTS-4, Bcl-2, cleaved caspase-3 (c-caspase3), Bax, IL-6, TNF-*α*, phosphorylated p65 (p-p65), p65, phosphorylated-AKT (p-AKT), AKT, phosphorylated-ERK (p-ERK), ERK, and GAPDH (1 : 5000, Proteintech, China) (Table 2), and secondary antibodies (1 : 5000, Proteintech, China). The protein bands were detected using a Tanon 5200 imaging system (Tanon, China).

### 2.8. Flow Cytometry

A flow cytometry assay was performed after the cells had been stained with propidium iodide (PI) and annexin V-FITC at 25°C for 20 min using the BD Biosciences Assay Kit (USA). A BD FACSCalibur Flow Cytometer (BD Biosciences, USA) was used for analysis.

### 2.9. JC-1 Assay

A Beyotime Biotechnology (China) assay kit was used for the JC-1 experiment. In brief, HNPCs were incubated with JC-1 staining solution at 37°C for 30 min and then washed three times using 1X JC-1 buffer.

### 2.10. Mitochondrial Permeability Transition Pore (Mptp) Assay

An Mptp Assay Kit (Beyotime Biotechnology) was used to investigate the opening of the Mptp. In brief, HNPCs were incubated with calcein AM staining solution at 37°C for 45 min and then with preheated culture solution at 37°C for 30 min, washed with PBS 2-3 times, and then added to detection buffer solution for observation under a fluorescence microscope.

### 2.11. ROS Assay

Dichlorodihydrofluorescein diacetate (DCFH-DA) was added to fresh serum-free medium at a dilution ratio of 1 : 1000 and added dropwise to the cell culture plate for cell incubation at 37°C for 20-30 min according to the instructions of the ROS assay kit (Beyotime Biotechnology) [37].

### 2.12. Histological Staining

Hematoxylin and eosin (HE) staining (G1120, Solarbio, China), Safranin O-fast green (SO-FG) staining (Servicebio, China), and Sirius red staining (G1018, Servicebio, China) were carried out following the manufacturer's procedures. A Pannoramic MIDI scanner (3DHISTECH, Hungary) was used to capture images.

### 2.13. Transmission Electron Microscopy (TEM)

The mitochondrial morphology of HNPCs was observed with a TEM instrument (HT7700; Hitachi, Japan) according to a previously described method [37].

### 2.14. Immunofluorescence Staining

Cells were immobilized, permeabilized, and incubated with the primary antibodies [37]. A concentration of 1 : 100 was used for the primary antibodies, including antibodies targeting iNOS, MMP13, COX-2, NLRP3, col-2, aggrecan, p65, and p-p65 (Table 2), and a concentration of 1 : 200 was used for the secondary antibody (ZSGB-Bio, China).

The sections were immersed in absolute ethanol for 5 min, 95% ethanol for 4 min, 90% ethanol for 3 min, 80% ethanol for 2 min, 70% ethanol for 2 min, and distilled water for 2 min. The sections were then washed 3 times with PBS for 5 min each, and 0.1% trypsin was used for antigen repair. The cells were washed 3 times with PBS for 3 min each, blocked with hydrogen peroxide blocking solution, and then washed again 3 times with PBS for 3 min each. Next, the cells were blocked with 5% bovine serum albumin (BSA) for 60 min, incubated with primary antibody at 4°C for 12 h, washed with PBS 3 times for 5 min each, incubated with secondary antibody at room temperature for 2 h, and washed with PBS 3 times for 5 min each. Nuclei were stained with DAPI and examined under a fluorescence microscope. A concentration of 1 : 100 was used for the primary antibodies, including antibodies targeting col-2, MMP13, IL-1*β*, COX-2, p-ERK, and p-AKT (Table 2), and a concentration of 1 : 200 was used for the secondary antibody (ZSGB-Bio, China). Images were captured with a Pannoramic 250 FLASH scanner (3DHISTECH, Hungary).

### 2.15. Statistical Analysis

The data are expressed as the mean ± standard deviation (SD) and were analyzed using GraphPad Prism v.5.0 software (GraphPad Software Inc., USA). Differences between groups were evaluated by one-way analysis of variance (ANOVA) with Tukey's post hoc test. A *p* value <0.05 was considered significant, and significance was denoted as follows: ^∗^*p* < 0.05, ^∗∗^*p* < 0.01, ^∗∗∗^*p* < 0.001, ^∗∗∗∗^*p* < 0.0001,^#^*p* < 0.05, ^##^*p* < 0.01 and ^###^*p* < 0.001.

## 3. Results

### 3.1. Effects of HT on HNPC Viability

**Figure 1:**
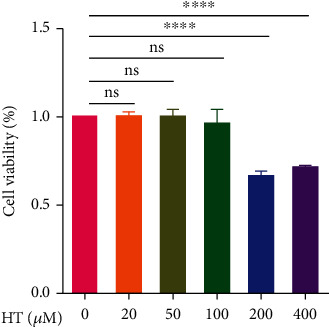
Effects of HT on HNPC viability. HNPCs were treated with different concentrations of HT (0, 20, 50, 100, 200, and 400 *μ*M) for 48 h, and cell viability was determined using a CCK-8 assay.

The results (Figure 1 showed that a cytotoxic effect of HT was not obvious at concentrations of 0, 20, 50, and 100 *μ*M. At the HT concentration of 100 *μ*M, cell viability was slightly decreased compared with that at the other concentrations, but there was no significant difference in viability between the 0 *μ*M and 100 *μ*M treatments. However, cell viability was significantly decreased at HT concentrations of 200 *μ*M and 400 *μ*M. Therefore, HT concentrations of 20 and 100 *μ*M were selected for the subsequent experiments.

### 3.2. HT Protects the Main Components of the IVD by Alleviating Inflammation and Mitigating ECM Degradation

**Figure 2:**
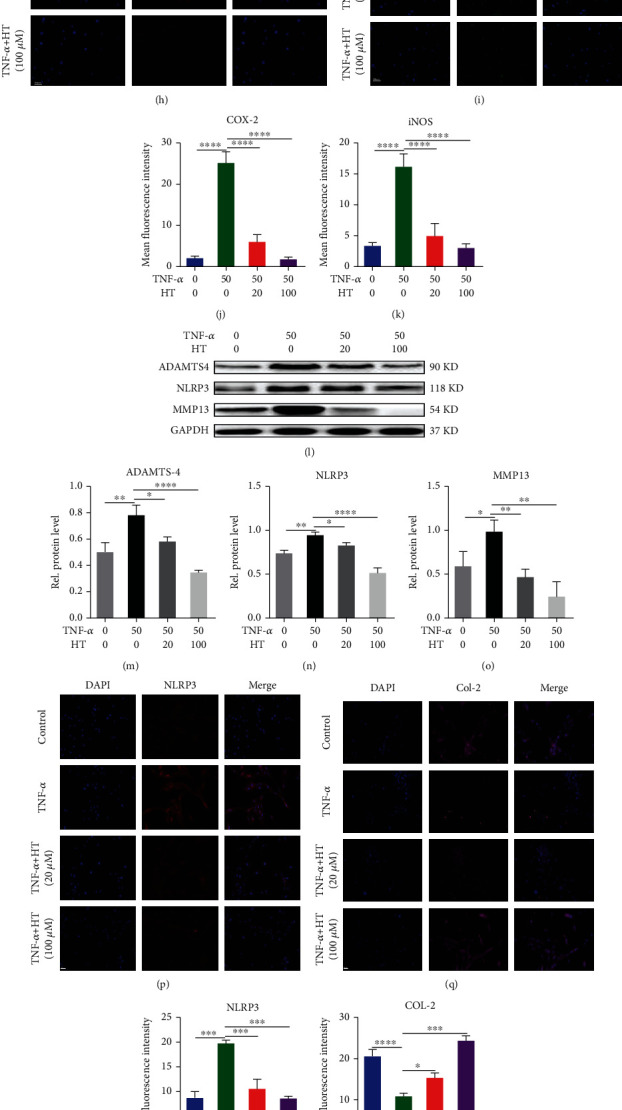
HT protects the main components of the IVD by alleviating inflammation and mitigating ECM degradation. Note: HNPCs were incubated for 24/48 h with 50 ng/mL TNF-*α* and 20 or 100 *μ*M HT or were left untreated. The expression of iNOS (a), COX-2 (b), MMP-13 (c), and ADAMTS-4 (d) was assayed by qRT–PCR. Protein levels of iNOS (e, f) and COX-2 (e, g) were assessed by WB. Immunofluorescence staining was used to assess the COX-2 (h, j), and iNOS (i, k) levels in HNPCs treated with TNF-*α* and HT. Scale bar: 50 *μ*m. Western blot detection for the expression of ADAMTS-4 (l, m), NLRP3 (l, n), and MMP13 (l, o) in each group. Immunofluorescence staining of NLRP3 (p, r), col-2 (q, s), aggrecan (t, w), and MMP-13 (u, v) in HNPCs. Scale bar: 50 *μ*m.

The release of inflammatory cytokines by NP cells is an important motile and pathological factor of IVDD. The increased secretion of ADAMTSs and MMPs is an important pathological process secondary to the inflammatory response, which can accelerate the imbalance of anabolism and catabolism in IVDs, destroy the homeostasis of the IVD, and aggravate IVDD. The qRT–PCR and WB results shown in Figure 2(a)-2(g)and 2(l)-2(o) reveal that after the HNPCs were stimulated with 50 ng/mL TNF-*α*, TNF-*α* induced increased protein and mRNA levels of iNOS, COX-2, MMP-13, ADAMTS-4, and NLRP3 in HNPCs, which promoted the degradation of IVD components. In contrast, 20 and 100 *μ*M HT treatment significantly inhibited the secretion of inflammatory cytokines that promote the occurrence and progression of IVDD. The immunofluorescence staining results shown in Figure 2(h)-2(k) and 2(p)-2(w) demonstrate that after TNF-*α* stimulation, the fluorescence intensity resulting from iNOS, COX-2, NLRP3 inflammasome, and MMP-13 staining was increased, indicating that the expression of these catabolism-promoting indicators was increased, and the fluorescence intensity resulting from col-2 and aggrecan staining was significantly decreased. HT treatment significantly reduced the secretion of inflammatory cytokines and significantly increased the fluorescence intensity resulting from col-2 and aggrecan staining.

### 3.3. HT Maintains Mitochondrial Homeostasis by Inhibiting the NF-*κ*B Signaling Pathway

**Figure 3:**
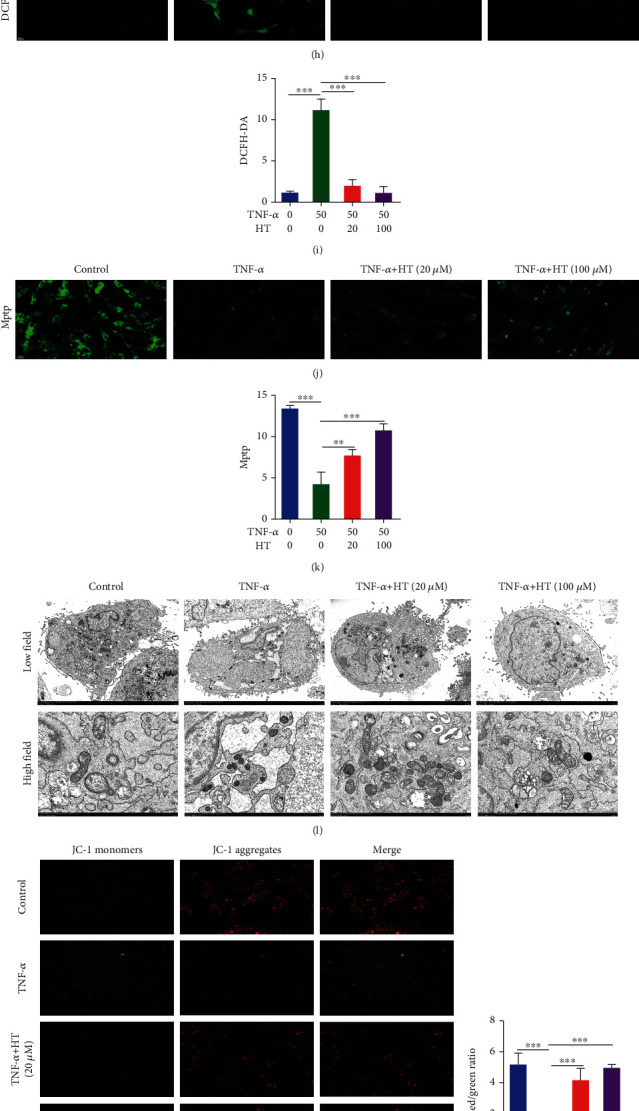
HT maintains mitochondrial homeostasis by inhibiting the NF-*κ*B signaling pathway. Note: HNPCs were incubated for 1 h with 50 ng/mL TNF-*α* and 20 or 100 *μ*M HT or were left untreated. (a) qRT–PCR measurement of NF-*κ*B_1_ levels in each group. (b, c) HNPCs were cultured according to the methods described above; Western blot measurement of p-p65 levels was performed. (d, f) The nuclear translocation rate of p65 was assessed by immunofluorescence staining. Scale bars: 25 *μ*m. (e, g). The expression level of p-p65 was measured using immunofluorescence staining. Scale bar: 50 *μ*m. HNPCs were incubated for 48 h with 50 ng/mL TNF-*α* and 20 or 100 *μ*M HT or were left untreated. (h, i) Endogenous ROS levels in HNPCs were measured with a DCFH-DA probe. Scale bar: 50 *μ*m. (j, k) Mptps in HNPCs were assayed by Mptp assay. Scale bar: 50 *μ*m. (l) Mitochondrial morphology was assessed by TEM. Scale bars: 5 *μ*m and 1 *μ*m. (m, n) The mitochondrial membrane potential in HNPCs was assessed by JC-1 staining. Scale bar: 50 *μ*m. (o) Flow cytometry was used to evaluate the apoptotic ratio of HNPCs in each group. (p) Western blot measurement of c-caspase3, Bax, and Bcl-2 levels in HNPCs was performed.

As illustrated in Figures 3(a)–3(g), following TNF-*α* treatment, the mRNA level of NF-*κ*B_1_ increased, a large amount of p65 was transferred from the cytoplasm to the nucleus, and the phosphorylation level of p65 in the nucleus was increased. After HT intervention, the mRNA level of NF-*κ*B_1_ and the nuclear translocation rate of p65 were significantly decreased, which decreased the expression level of p-p65. As illustrated in Figures 3(h)–3(k), 50 ng/mL TNF-*α* significantly increased the endogenous ROS content in HNPCs in vitro. Subsequent Mptp experiments revealed that TNF-*α* led to the opening of the Mptp, which damaged mitochondrial function and triggered mitochondrial apoptosis. HT intervention significantly reduced the increase in endogenous ROS levels induced by TNF-*α* and protected mitochondrial function by reducing Mptp opening. As shown in Figure 3(l), we observed the morphology of mitochondria directly by TEM. After TNF-*α* treatment, the morphology of HNPC mitochondria was damaged, the mitochondria had become swollen, and the mitochondrial crest had disappeared, which indirectly indicated mitochondrial dysfunction in HNPCs. Moreover, the change in mitochondrial membrane potential was evaluated by JC-1, and the results are shown in Figures 3(m) and 3(n). We found that TNF-*α* caused a decrease in the mitochondrial membrane potential of HNPCs, as indicated by a decreased red/green fluorescence value, suggesting that normal mitochondrial function was damaged. In contrast, TNF-*α*-induced pathological changes were significantly mitigated after HT treatment. Assessments of mitochondrial morphology showed that HT reduced the number of mitochondria with abnormal morphology, reduced the opening of the Mptp, and alleviated damaged mitochondrial function by improving mitochondrial membrane potential. As shown in Figures 3(o) and 3(p), TNF-*α* induced apoptosis in 13.4% of HNPCs, whereas HT significantly reduced the apoptosis rate of HNPCs: the apoptosis rate of HNPCs treated with 20 *μ*M HT was 8.49%, and that of HNPCs treated with 100 *μ*M HT was 5.18%. After TNF-*α* treatment, the expression of c-caspase3 and Bax (employed as a proapoptotic evaluation index) in HNPCs increased, while the expression of Bcl-2 (serving as an antiapoptotic evaluation index) decreased. HT treatment decreased the production of proapoptotic indicators and increased the secretion of antiapoptotic indicators.

### 3.4. HT Alleviates IVDD in a Rat Needle Puncture Model In Vivo

**Figure 4:**
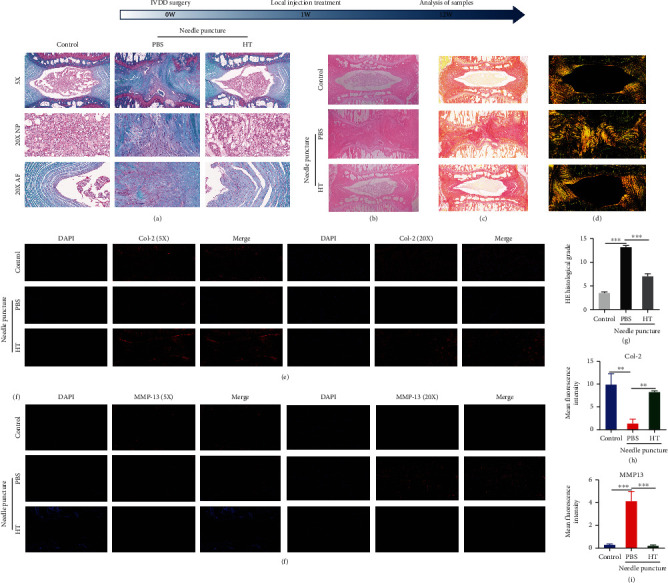
HT alleviates IVDD in a rat needle puncture model in vivo. (a) SO-FG was used to measure the proteoglycan contents in the IVDs of the rats in each group; scale bars: 200 *μ*m and 50 *μ*m. (b, g) HE staining was performed for the histopathological analysis of IVD morphology in each group; scale bar: 250 *μ*m. (c, d) Sirius red staining and polarized light microscopy assessments of collagen; scale bar: 250 *μ*m. Immunofluorescence staining was used to measure the levels of col-2 (e, h) and MMP-13 (f, i); scale bars: 200 *μ*m and 50 *μ*m. *N* = 5.

The HE staining results in Figures 4(b) and 4(g) show that the caudal space in rats was narrowed after puncture. Sagittal pathological staining of the IVD revealed structural disorder, and the structures of the NP and AF could not be distinguished. HT injection mitigated the degeneration of the IVD in rats. The pathological results showed that the two adjacent spaces in the rat caudal vertebra were wider in the HT-treated rats than in the puncture-only rats, and the structure of the NP and AF could be clearly distinguished in the HT-treated rats. As shown in Figure 4(a), SO-FG staining revealed a significantly reduced content of proteoglycans in the degenerative IVD of rats, and the loss of proteoglycans was alleviated by HT treatment. As illustrated in Figures 4(c) and 4(d), picrosirius red staining and polarized light results showed that HT treatment significantly mitigated the loss of collagen content in rat IVDs caused by needle puncture. As illustrated in Figures.  4(e)–4(i), HT reduced MMP-13 expression, thereby preventing the loss of col-2 expression caused by excessive catabolic enzyme production.

### 3.5. HT Inhibits the LPS-Induced Microglial Inflammatory Response

**Figure 5:**
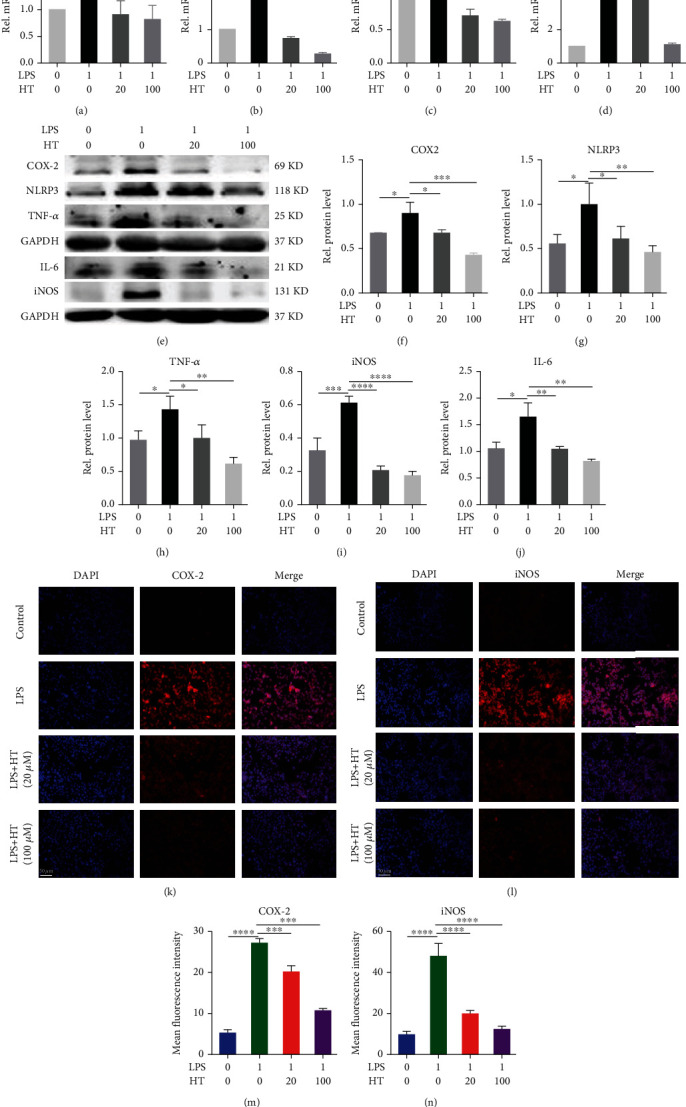
HT reduces the LPS-induced microglial inflammatory response. Note: HNPCs were incubated for 24/48 h with 1 *μ*g/mL LPS and 20 or 100 *μ*M HT or were left untreated. After LPS and HT stimulation, the levels of IL-1*β* (a), IL-6 (b), COX-2 (c), and iNOS (d) were measured using qRT–PCR, and the expression levels of COX-2 (e, f), NLRP3 (e, g), TNF-*α* (e, h), IL-6 (e, j), and iNOS (e, i) in microglia were determined by WB. Representative immunofluorescence staining images of the COX-2 (k, m), and iNOS (l, n) levels in each group are shown. Scale bar: 50 *μ*m.

To assess the pathological process, we examined the mechanism by which HT reduces the inflammatory response of microglia to identify a new treatment method for relieving neuropathic pain. As shown in Figures 5(a)–5(d), microglial stimulation with 1 *μ*g/mL LPS resulted in the release of inflammatory cytokines and increased the levels of secreted IL-1*β*, IL-6, COX-2, and iNOS mRNA. The expression of IL-1*β*, IL-6, COX-2, and iNOS in microglia was significantly inhibited after HT stimulation. As shown in Figures 5(e)–5(j), LPS treatment increased the inflammatory response of microglia, resulting in increased secretion of COX-2, NLRP3, TNF-*α*, IL-6, and iNOS. However, HT treatment inhibited this pathological process by reducing the expression of these inflammatory cytokines. As shown in Figures 5(k)–5(n), immunofluorescence staining revealed that HT decreased the fluorescence intensity resulting from COX-2 and iNOS staining, indicating that HT played a role in maintaining the inflammatory homeostasis of microglia.

### 3.6. HT Alleviates Neuropathic Pain and Reduces the Inflammatory Response in Rats after CCD

**Figure 6:**
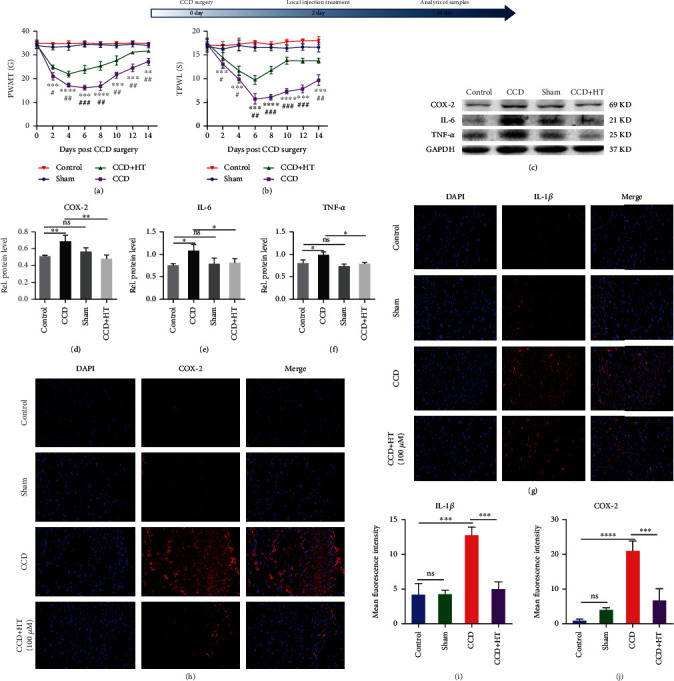
HT alleviates neuropathic pain and reduces the inflammatory response in rats after CCD. (a) PWMT was calculated every 2 days after CCD to assess the mechanical pain in the ipsilateral posterior claw of rats. *N* = 5. ^∗^CCD group vs. Control group, ^#^ CCD group vs. CCD + HT group. (b) TPWL was calculated every 2 days after CCD to assess the pain in the ipsilateral posterior claw of rats. *N* = 5. (c–f) The expression levels of COX-2 (c, d), IL-6 (c, e), and TNF-*α* (c, f) in the SDH of rats were determined by WB. *N* = 5. Representative immunofluorescence staining images of IL-1*β* (g, i) and COX-2 (h, j) staining are shown. *N* = 5, scale bar: 20 *μ*m.

We further explored the role of HT in alleviating neuropathic pain in rats by establishing a rat model of CCD and intrathecally administering HT. PWMT and TPWL (behavioral evaluation indices) were measured in the rats every two days beginning on the second day after surgery. The PWMT and TPWL values in the rats in the CCD model group continuously decreased from the 2nd day to the 8th day after surgery and began to recover on the 8th day after surgery. As illustrated in Figures 6(a) and 6(b), HT was injected on the second day after the establishment of the CCD model. The PWMT and TPWL values of the rats had improved beginning on day 2 after HT injection and continued to improve through day 14.

Inhibiting the expression of various proinflammatory cytokines in microglia ameliorates the worsening of neuropathic pain. As illustrated in Figures 6(c)–6(f), the protein levels of COX-2, IL-6, and TNF-*α* in the SDH of rats increased after CCD. As illustrated in Figures.  6(g)–6(j), immunofluorescence staining showed that HT decreased the fluorescence intensity resulting from IL-1*β* and COX-2 staining. Notably, HT reduced the production of inflammatory cytokines in the SDH after CCD, indicating that it played a role in alleviating neuropathic pain.

### 3.7. HT Alleviates Neuropathic Pain by Inhibiting the PI3K/AKT and ERK Signaling Pathways

**Figure 7:**
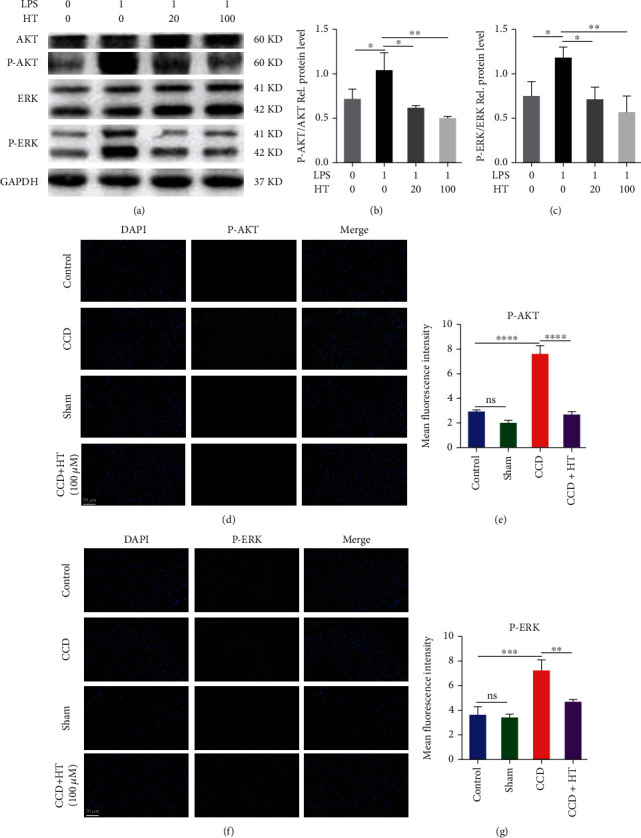
HT alleviates neuropathic pain by inhibiting the PI3K/AKT and ERK signaling pathways.

As shown in Figures.  7(a)–7(c), LPS stimulation activated the PI3K/AKT and ERK signaling pathways by increasing the levels of p-ERK and p-AKT in microglia. In addition, HT inhibited PI3K/AKT and ERK signaling pathway activation by decreasing the p-ERK and p-AKT levels. In verifying our experimental results, we found that the expression of p-ERK and p-AKT increased in rats after CCD, suggesting that the PI3K/AKT and ERK signaling pathways are involved in the development of neuropathic pain. However, intrathecal injection of HT significantly reduced the expression levels of p-ERK and p-AKT in the SDH of rats. These findings indicate that intrathecal injection of HT inhibited the activation of the PI3K/AKT and ERK signaling pathways by reducing the levels of p-ERK and p-AKT in the SDH (Figures 7(d)–7(g)).

HNPCs were incubated for 2 h with 1 *μ*g/mL LPS and 20 or 100 *μ*M HT or were left untreated. (a–c) Western blot analysis of AKT, p-AKT, ERK, and p-ERK expression in HNPCs. (d–g) Representative immunofluorescence staining images of p-AKT and p-ERK expression in the SDH in the control group, sham group, CCD group, and CCD group after HT treatment were obtained by immunofluorescence staining. *N* = 5, scale bar: 50 *μ*m.

Specific mechanisms by which HT alleviates IVDD and neuropathic pain. HT ameliorates the inflammatory response and ECM degeneration, reduces the apoptosis rate, alleviates mitochondrial dysfunction, and maintains IVD homeostasis by inhibiting the NF-*κ*B signaling pathway. HT reduces the inflammatory response and ameliorates neuropathic pain by inhibiting the PI3K/AKT and ERK signaling pathways.

## 4. Discussion

IVDD is caused by multiple pathological factors, including genetic factors, trauma, excessive mechanical loads, aging, and smoking [40]. In response to various pathological factors, the expression of inflammatory cytokines and chemokines is upregulated in IVD cells [6, 41, 42]. Subsequently, the expression of the catabolic molecules ADAMTSs and MMPs is increased in IVD cells [43, 44]. Catabolic molecules from NP and AF cells promote ECM degradation. Therefore, inhibition of the inflammatory responses and IVD catabolism is important for alleviating IVDD. TNF-*α* is commonly utilized to simulate the local microenvironment. In the current study, HT improved the inflammatory response of HNPCs, alleviated the degradation of the ECM, and significantly inhibited the loss of major components of the IVD. Therefore, HT balances the relationship between catabolism and anabolism in the IVD and inhibits the loss of major IVD components by inhibiting the expression of enzymes that promote catabolism. In addition, TNF-*α* plays an important role in mediating ECM degradation by inducing NLRP3 inflammasome activation [45]. In the current study, HT significantly reduced the increase in the expression of the NLRP3 inflammasome observed during IVDD, thereby ameliorating ECM degradation.

The NF-*κ*B signaling pathway triggers multiple pathological processes that lead to IVDD, including the inflammatory response, matrix degradation, and imbalance in mitochondrial homeostasis [45]. Therefore, we aimed to explore the potential regulatory mechanism of HT in the inhibition of the NF-*κ*B signaling pathway in the pathological process of IVDD and thereby identify appropriate therapeutic targets for improving or even blocking IVDD. In our study, TNF-*α* was found to increase the expression of NF-*κ*B_1_, and TNF-*α* induced the phosphorylation of p65 in the nucleus by increasing its nuclear translocation rate, thereby increasing the expression of p-p65 protein. Our current study demonstrated that HT inhibits the TNF-*α*-induced activation of the NF-*κ*B signaling pathway by inhibiting p65 nuclear translocation and phosphorylation.

An imbalance in mitochondrial homeostasis can lead to abnormal increases in ROS and secondary oxidative stress reactions as well as decreases in mitochondrial membrane potential. These phenomena disrupt the homeostasis of energy metabolism in the IVD, resulting in an imbalance of anabolism and catabolism, and subsequently causing the apoptosis of NP cells and exacerbating IVDD progression [45]. Our results showed that HT can significantly inhibit the activation of the NF-*κ*B signaling pathway. Moreover, HT reduced the opening of the Mptp and the loss of mitochondrial membrane potential by reducing the production of ROS in HNPCs caused by TNF-*α*, which was verified by assessments of mitochondrial morphology. HT treatment significantly inhibited the increase in the proapoptotic index and increased the antiapoptotic index. Therefore, HT can reduce apoptosis in HNPCs by maintaining mitochondrial homeostasis during IVDD.

Next, we used a rat needle puncture degeneration model to evaluate the role of HT in alleviating IVDD. HT can alleviate stenosis in the caudal space caused by needle puncture, prevent col-2 loss, and reduce MMP-13 expression. Thus, HT maintains IVD homeostasis by inhibiting the pathogenesis of the IVD.

With the exacerbation of IVDD, the NP may herniate and compress the dural sac and surrounding nerve tissue, thereby inducing neuropathic pain [16]. Therefore, much effort is being made to identify drugs that can relieve IVDD and neuropathic pain to improve the symptoms of LBP and the quality of life of patients. Because the IVD is a tissue severely lacking blood supply [46], local puncture injection is an important means of delivering drugs to NP cells. Since the dural sac is posterior to the IVD, we envision delivering the drug to the dural sac by a single puncture to relieve neuropathic pain and then adjusting the direction of the puncture needle to deliver the drug into the NP tissue to relieve IVDD. Therefore, a single puncture can solve these two clinical problems, reduce pain in patients and the treatment cost, and improve the treatment effect.

Microglia in the SDH release a variety of inflammatory cytokines that initiate neuropathic pain [47]. Therefore, inhibition of the inflammatory response in the SDH is an important method for alleviating neuropathic pain. In the rat CCD model of the present study, the secretion of proinflammatory cytokines in the SDH was increased. Intrathecal injection of HT significantly antagonized the expression of inflammatory cytokines in the SDH after CCD. In vitro experiments showed that HT also significantly reduced the levels of inflammatory cytokines in microglia to relieve neuropathic pain.

The PI3K/AKT and ERK signaling pathways play important roles in the development of neuropathic pain. Inhibition of PI3K/AKT and ERK signaling pathway activation is an important approach for treating neuropathic pain. ERK signaling pathway activation leads to the production of various cytokines, including TNF-*α*, IL-1*β*, COX-2, and iNOS, that are involved in the enhancement of neuropathic pain [48–50]. The PI3K/AKT pathway modulates nociceptive information and mediates central sensitization induced by noxious stimuli [51, 52]. Inhibition of PI3K/AKT significantly reduces inflammation and neuropathic pain [53, 54]. Our cell and animal experiments showed that HT plays an important role in inhibiting the ERK and AKT signaling pathways by reducing the expression of p-ERK and p-AKT.

## 5. Conclusions

**Figure 8:**
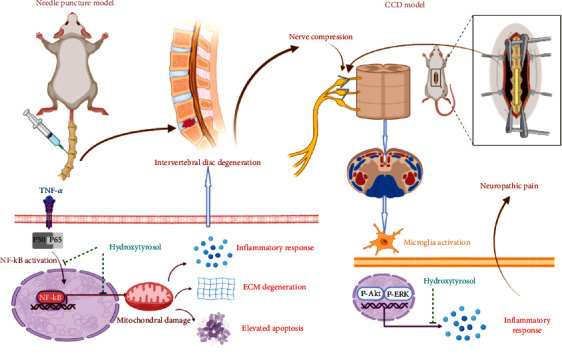
Schematic diagram showing that HT alleviates IVDD and neuropathic pain.

In conclusion, HT inhibits inflammatory responses, attenuates oxidative stress, and ameliorates mitochondrial function to maintain IVD homeostasis by inhibiting the NF-*κ*B signaling pathway. HT alleviates neuropathic pain by inhibiting the PI3K/AKT and ERK signaling pathways. Therefore, HT ameliorates IVDD and its secondary neuropathic pain. These findings suggest a new approach for treatment (Figure 8).

## Figures and Tables

**Table 1:** The nucleotide sequences of the primers used in this experiment.

Source	Target	Forward primer, 5′-3′	Reverse primer, 5′-3′
Human	COX-2	GGAACTTTCTGGTCCCTTCAG	TGTGTTTGGAGTGGGTTTCA
	iNOS	GCCAAGCTGAAATTGAATGAGGA	TTCTGTGCCGGCAGCTTTAAC
	MMP-13	TGCTGCATTCTCCTTCAGGA	ATGCATCCAGGGGTCCTGGC
	ADAMTS-4	ACCCAAGCATCCGCAATC	CAGGTCCTGACGGGTAAACA
	NF-*κ*B_1_	TATTTGAAACACTGGAAGCACG	CCGGAAGAAAAGCTGTAAACAT
	GAPDH	GCACCGTCAAGGCTGAGAAC	TGGTGAAGACGCCAGTGGA

Rat	COX-2	CTACACCAGGGCCCTTCC	TCCAGAACTTCTTTTGAATCAGG
	iNOS	CACCACCCTCCTTGTTCAAC	CAATCCACAACTCGCTCCAA
	IL-6	AAGCCAGAGTCATTCAGAGCAA	GGTCCTTAGCCACTCCTTCT
	IL-1*β*	AAATGCCTCGTGCTGTCTGA	CAAGGCCACAGGGATTTTGTC
	GAPDH	CCACCAACTGCTTAGCCCCC	GCAGTGATGGCATGGACTGTGG

**Table 2:** Antibodies used in this experiment.

Primary antibody	Catalog number	Manufacturer
Anti-NLRP3	A5652	ABclonal
Anti-COX-2	A1253	ABclonal
Anti-MMP13	A11148	ABclonal
Anti-iNOS	A14031	ABclonal
Anti-ADAMTS-4	BS-4191R	Bioss
Anti-Bcl-2	A1105	ABclonal
Anti-cleaved caspase-3	9664	Cell signaling technology
Anti-Bax	A19684	ABclonal
Anti-IL-6	A2447	ABclonal
Anti-TNF-*α*	60291-1-Ig	Proteintech
Anti-phosphorylated p65 (anti-p-p65)	AF2006	Affinity
Anti-p65	3033	Cell signaling technology
Anti-phosphorylated AKT (anti-p-AKT)	9271	Cell signaling technology
Anti-AKT	9272	Cell signaling technology
Anti-phosphorylated ERK (anti-p-ERK)	9101	Cell signaling technology
Anti-ERK	9102	Cell signaling technology
Anti-GAPDH	10494-1-AP	Proteintech
Anti-col-2	AF0135	Affinity
Anti--aggrecan	DF7561	Affinity
Anti-IL-1*β*	A19635	ABclonal

## Data Availability

Data are available from the corresponding author upon request.

## Abbreviations

LBP: Low back pain
IVDD: Intervertebral disc degeneration
IVD: Intervertebral disc
LPS: Lipopolysaccharide
COX-2: Cyclooxygenase-2
iNOS: Inducible nitric oxide synthase
NLRP3: NOD-like receptor thermal protein domain associated protein 3
NF-*κ*B: Nuclear factor kappa-B
PI3K: Phosphatidylinositol 3-kinase
AKT: Protein kinase B
ERK: Extracellular regulated protein kinase
ADAMTS: A disintegrin and metalloproteinase with thrombospondin motifs
MMP: Matrix metalloproteinase
ROS: Reactive oxygen species
TNF-*α*: Tumor necrosis factor-*α*
IL: Interleukin
Col-2: Collagen-2
NP: Nucleus pulposus
AF: Annulus fibrosus
HNPC: Human nucleus pulposus cell
HT: Hydroxytyrosol
JOA: Japanese Orthopaedic association
SD: Standard deviation
DCFH-DA: Dichlorodihydrofluorescein diacetate
ECM: Extracellular matrix
HE: Hematoxylin and eosin
SO-FG: Safranin O and fast green staining
TEM: Transmission electron microscopy
c-caspase3: Cleaved caspase-3
p-p65: Phosphorylated p65
p-AKT: Phosphorylated AKT
p-ERK: Phosphorylated ERK
SDH: Spinal dorsal horn
CCD: Chronic compression of the dorsal root ganglion
PWMT: Paw withdrawal mechanical threshold
TPWL: Thermal paw withdrawal latency
qRT–PCR: Quantitative real-time PCR
Mptp: Mitochondrial permeability transition pore.

## Additional Points

*Institutional Review Board Statement.* The study was approved by the Institutional Ethics Review Board of Chinese PLA General Hospital (2021-323-01). The experimental protocol was established according to the guidelines of the Manipulative Technique for the Care and Use of Laboratory Animals, China, and approved by the Animal Ethical and Welfare Committee of Chinese PLA General Hospital.
